# How peer conversations about HIV/AIDS media messages affect comprehension and beliefs of young South African women

**DOI:** 10.1080/17290376.2016.1197146

**Published:** 2016-06-16

**Authors:** E. Lubinga, A.A. Maes, C.J.M. Jansen

**Affiliations:** ^a^ PhD, is a Senior Lecturer in Communication Studies in the Department of Communication, Media and Information Studies, University of Limpopo, Sovenga, Limpopo, South Africa; ^b^ PhD, is a Full Professor, and Head of the Department of Communication and Information Sciences, Tilburg University, Tilburg, The Netherlands; ^c^ PhD, is a Full Professor, and Head of the Department of Communication and Information Studies, University of Groningen, The Netherlands. He is also affiliated with the Language Centre at Stellenbosch University, Stellenbosch, South Africa

**Keywords:** beliefs, comprehension, health messages, interpersonal discussions, female adolescents, prevention, Convictions, Compréhension, Messages de santé, Discussions interpersonnelles, Adolescentes, Prévention

## Abstract

Most existent research on the effects of interpersonal discussions about health campaign messages is based on surveys. In this study, we analysed actual conversations about an HIV/AIDS poster to find out possible effects. Young South African women in 59 dyads (*n* = 118) participated in conversations about a deliberately puzzling HIV and AIDS poster that cautioned the target group to be faithful to one sexual partner. We measured their comprehension of the poster and beliefs about the message, before and after the conversations. Overall, actual comprehension (AC) was low, and we observed a large discrepancy between actual and perceived comprehension. In general, conversations did not improve AC. It proved to be even more probable that a correct interpretation before a conversation turned into an incorrect interpretation than the other way around. However, having a well-informed conversation partner increased the chance of acquiring adequate subsequent comprehension. We found, in general, that conversations did not decrease undesirable beliefs. One important undesirable belief even became reinforced after the conversations. Conversations among peers might be valuable in health campaigns, but our study shows that intended positive effects do not automatically follow.

## Résumé

La plupart des recherches existantes sur les effets des discussions interpersonnelles concernant des messages de campagne de santé est basée sur des enquêtes. Dans cette étude, nous avons analysé des conversations réelles autour d'une affiche VIH/SIDA pour en découvrir les possibles effets. Cinquante-neuf dyades (*n* = 118) de jeunes femmes sud-africaines ont participé à des conversations autour d'une affiche volontairement troublante sur le VIH/SIDA qui conseillait au groupe cible d'être fidèle à un partenaire sexuel. Nous avons mesuré leur compréhension de l'affiche et leurs convictions sur le message, avant et après les conversations. Dans l'ensemble, la compréhension réelle était faible, et nous avons observé un grand écart entre la compréhension réelle et perçue. En général, les conversations n'ont pas amélioré la compréhension réelle. Il est apparu qu'il était même plus probable qu'une interprétation correcte avant une conversation soit transformée en une interprétation incorrecte que l'inverse. Cependant, le fait d'avoir un partenaire de conversation bien informé a augmenté les chances d'acquérir une compréhension ultérieure adéquate. Nous avons trouvé en général que les conversations ne réduisaient pas les convictions indésirables. Une conviction indésirable importante est même sortie renforcée des conversations. Les conversations entre pairs peuvent être précieuses dans les campagnes de santé, mais notre étude montre que les effets positifs attendus ne suivent pas automatiquement.

## Research problem

Most existent research on the effects of interpersonal discussions about health campaign messages is based on surveys. In the present study, we analyse the content and quality of conversations induced by deliberately puzzling health messages. In particular, we investigate the effects of such discussions on the actual comprehension of the participants (do they understand the message?), on their perceived comprehension (do they think they understand the message?) and on their beliefs regarding the topic of the health message.

## 1. Introduction

Researchers, through empirical studies, have established that the efficacy of mass media health communication campaigns can be greatly enhanced by conversations about the core messages of these campaigns. Conversations about health messages can, for example, lead to changes in relevant beliefs (Geary, Burke, Castelnau, Neupane, Sall, Wong *et al*. 2007; Hwang 2012), attitudes (Dunlop, Kashima & Wakefield 2010; Hendriks, Van den Putte & De Bruijn 2014a), social norms (Chatterjee, Bhanot, Frank, Murphy & Power 2009; Dunlop, Cotter & Perez 2014; Kam, Potocki & Hecht 2012) and behavioural intentions (Busse, Fishbein, Bleakley & Hennessy 2010; Reimuller, Hussong & Ennett 2011; Van den Putte, Yzer, Southwell, De Bruijn & Willemsen 2011), thus fostering behaviour change.

Two theories are particularly relevant in explaining when and why target audiences engage in interpersonal discussions about health-related media campaign messages (‘conversational occurrence’, cf. Hendriks, Van den Putte, De Bruijn & De Vreese 2014b:626). The Two-Step Flow Theory, developed by Katz and Lazarsfeld (1955) suggests that there are networks of interconnected individuals through which media messages are channelled. From the pattern of information flow, two steps are distinguished. In step one, the opinion leaders who are the more regular users of the media receive the information from the media. In a second step, these opinion leaders interpret the content, and pass it on to secondary, less frequent media users, who might in turn be influenced by the message. This theory predicts the effects of media messages, specifically the influence of interpersonal relationships on decision making among individuals. The Diffusion of Innovations Theory by Rogers (1995) asserts that interpersonal networks are crucial to the rapid diffusion of new ideas. These networks create awareness about new ideas and their source, and result in persuasion through peer–peer or near–peer interaction.

A number of researchers have investigated how message exposure is related to conversational occurrence, and how conversations in turn, are related to behaviour or behavioural intentions (Busse *et al*. 2010; Hendriks *et al*. 2014b; Hornik & Yanovitsky 2003; Hwang 2012; Southwell 2005). One of the findings is that the frequency of conversations is positively related to health behaviour. For example, Dillorio, Kelly and Hockenberry-Eaton (1999) found that the frequency of sexuality communication was associated with delayed initiation of sexual relations among adolescents. Lefkowitz, Boone, and Shearer (2004) found that more frequent and comfortable conversations among college students about the dangers of sexual behaviour were generally associated with more positive condom-related attitudes.

Health behaviour is not only associated with conversational occurrence but also with the nature of the conversation process (Arroyo & Harwood 2012; Dunlop *et al*. 2010). One type of studies focuses on the way people talk, negatively or positively, about campaign messages (‘conversational valence’, cf. Hendriks *et al*. 2014a:684). For example, in a study by Hendriks *et al*. (2014a) participants reported more negative conversations about binge drinking when they were exposed to an anti-alcohol message. Furthermore, as conversations about alcohol consumption became more negative, the participants’ intentions to refrain from binge drinking increased (684). Dunlop (2011) found that smokers who talked positively about a campaign message reported beliefs, attitudes and intentions that were more in agreement with the message. Khalil and Rintamaki (2014) conducted a survey among 1325 participants about a televised entertainment education drama intended to promote positive discussions about organ donation. They conclude that including accurate information may be effective in myth rejection and promoting positive discussions about organ donation.

## 2. Studying the effects of deliberately puzzling messages on conversation intention

In recent studies, a number of message features have been suggested to stimulate conversations, such as the use of narratives (Dunlop *et al*. 2010; Frank, Murphy, Chatterjee, Moran & Baezconde-Garbanati 2015), the use of new technologies (Baelden, Audenhove & Vergnani 2012) and the use of emotional appeals (Hafstad & Aaro 2009; Nabi 2015). In this study, we focus on the deliberate use of puzzling elements in health campaign messages. Both the South African health organisation loveLife (in Hollemans 2005) and Hoeken, Swanepoel, Saal, and Jansen (2009) suggest, albeit on different grounds, that including puzzling elements in health messages could spark conversations about these messages. From 2000 until 2009, loveLife used deliberately puzzling messages in their health promotion campaigns. The assumption was that if the audience found the message difficult to understand they would want to talk about it to improve their comprehension. As stated by loveLife’s media director Mandla Ndlovu in a radio interview from 2006 (quoted in Robbins 2010): ‘The billboards are there to spark discussion and thought among people, among women, among men, among everybody. What exactly do the loveLife billboards mean? Ask your friends’ (226).

Hoeken *et al*. (2009) built on this assumption and developed a theoretical model derived through a review of literature on the effects of tropes such as metaphors, ellipses and other deliberately puzzling expressions, on conversations and campaign outcomes. Hoeken *et al*. started from the notion of perceived comprehension (PC) by the recipients. Inspired by group dynamic processes as studied in an ethnographic study by Ritson and Elliot (1999) on advertisements in the model, Hoeken *et al*. hypothesise two ways in which deliberately puzzling messages might stimulate conversations among young recipients. A first possibility is that, on exposure to such a message, recipients think that they themselves understand the message whereas others do not. They might then start a conversation, to show off and impress their peers who presumably do not understand the message. A second possibility is that recipients think that they and their peers understand the message. They might then start conversations to create a degree of togetherness with their peers, at the same time distancing themselves from outsiders like elder people, parents or teachers, who are assumed not to understand the message.

Hoeken *et al*. (2009) further suggest possible undesirable side effects of the use of puzzling messages, such as yielding incomprehension or miscomprehension, resulting in the priming of unintended, possibly even dangerous beliefs that might influence global perceptions, behavioural intentions, and ultimately behaviour in a negative way (Fishbein & Yzer 2003). In a typology on the unintended effects of health communication campaigns, Cho and Salmon (2007:298) state that one of the most common unintended effects is obfuscation: the creation of confusion and misunderstanding of health risks and risk prevention methods, attributable to limitations in message design and delivery.

In an attempt to test the assumptions by Hoeken *et al*. (2009) and by loveLife (Hollemans 2005), we tried to find out, in four earlier studies, whether and if so, under what conditions conversations about puzzling health messages are likely to occur (Jansen & Janssen 2010; Lubinga & Jansen 2011; Lubinga, Jansen & Maes 2014; Lubinga, Schulze, Jansen & Maes 2010). The results of these studies contradict the predictions by loveLife, and also by Hoeken *et al*. (2009). The necessary condition for willingness to engage in a conversation was not found to be a perceived own lack of comprehension, as loveLife assumes, nor was it perceived own comprehension combined with perceived lack of comprehension in others, as Hoeken *et al*. (2009) assume. Rather, the outcomes so far suggest that it is a combination of perceived own comprehension of the puzzling messages, PC by the conversation partner, perceived relevance of the message, and appreciation of the message, that might have a positive influence on the willingness to talk about puzzling messages. Our studies also show some of the negative consequences of puzzling messages as proposed by Hoeken *et al*. (2009) and identified by Cho and Salmon (2007), especially the occurrence of wrong or dangerous interpretations of intended messages. For example, one of the young women wrongly interpreted a message by loveLife, ‘Prove your love, protect me’ as ‘Having sex with him means that you will be proving your love for him’ (Lubinga *et al*., 2010:182). Similar misinterpretations of another loveLife message are observed by Singer (2005), quoting a study commissioned by loveLife in which 19–62% of students (depending on the advert) reportedly understood the messages. That study showed that those who were considered to be highly at risk in terms of contracting HIV and AIDS – rural, poor and black students – found it most difficult to understand the messages. In one case, an advert was erroneously interpreted as ‘You must pressurise, force the girl to have sex with you’ by a group of teens.

## 3. Studying conversations about health-related topics

The research method used in our previous studies was similar to what is done in many other studies in this field (Busse *et al*. 2010; Dunlop, Wakefield & Kashima 2008; Frank, Chatterjee, Chaudhuri, Lapsansky, Bhanot, Murphy *et al*. 2012; Geary et. al., 2007; Helme, Noar, Allard, Zimmerman, Palmgreen, McClanahan *et al*. 2011). Participants were asked to study and interpret messages and were presented with a questionnaire in which they were asked about their potential conversation behaviour. That type of data, however, does not allow for firm conclusions about the extent to which the messages would be discussed in reality, and even less so about the quality of such conversations. As Southwell and Yzer (2009:6) state,

[ … ] communication scholars who bother to include interpersonal conversation in their models have tended to avoid the specific issue of accounting for what is actually said and essentially to treat such variation as noise. Nonetheless, it may well be that investigation of content-related contingencies is where we need to go next.

Hendriks *et al*. (2014b) draw the same conclusion and state that, ‘Future researchers should explore the role of conversational content in more detail’ (634).

In some studies, researchers have already focused on the quality of conversations on health-related issues by recording and observing actual conversations. For example, Boone and Lefkowitz (2007) observed the communication strategies used by mothers in discussing various health topics with their adolescent children. The analysis of 52 mother-adolescent dyads revealed that in conversations about drugs/alcohol, sexuality and nutrition/exercise, the mothers spent most of the time asking questions, rather than discussing the negative consequences or lecturing the adolescents. In another study by Lefkowitz *et al*. (2004) about peer communication with best friends, not only conversation quality was studied, but also how this affects the adolescent’s behaviour. They concluded that adolescents influence each other’s behaviour through their conversations, feel more comfortable talking to peers and find information from peers more useful than from parents.

More recently, Hendriks *et al*. (2014b) set up and observed conversations about a no-alcohol and an anti-alcohol message, in a laboratory situation. Their intention was to predict the relation between message exposure, conversation occurrence and behaviour change. They monitored the conversations to ensure that the participants stayed on course while talking about the topic they were asked to discuss (629). No mention is made of an analysis of the content, or the quality of the conversations.

With the present study, we aim to gain insight into the effects of interpersonal discussions induced by deliberately puzzling health messages by examining the content of conversations by the participants. In particular, we wish to investigate the effects of such discussions on the actual comprehension (AC; do they understand the message?), of the participants; on their PC (do they think they understand the message?) and on their beliefs regarding the topic that the health message focuses on. Furthermore, we are interested in the possible influence of AC and PC before they started their conversation on the quality and the quantity of their contributions to the conversations.

For this purpose, we collected 59 dyadic peer conversations between young women discussing an HIV and AIDS poster about the risks involved in sharing sexual partners, and we carried out an analysis of the content of all information units. We measured the participants’ AC and PC, and also their beliefs with respect to sharing sexual partners, before and after the conversation.

## 4. Methods

### 4.1. Design of the study

We asked a total of 59 dyads (*n* = 118) of young women to hold a conversation about their interpretation of the meaning of an HIV and AIDS poster with a deliberately puzzling message. We used a questionnaire, both before and after the conversation, to measure their comprehension of and beliefs about the poster message. The conversations, carried out in English, took place in four rural secondary schools in one of the South African Provinces. In each school, the following three phases of the experiment were scheduled on the same regular school day.

- *Phase 1*: We presented 50 young women per school with a poster on multiple concurrent sexual partnerships and the spread of HIV and AIDS. In order to determine their level of AC and PC, as well as their beliefs regarding the theme on the poster, we asked them to fill in a questionnaire.
- *Phase 2*: After this, we asked a selection of the participants to have a conversation about the meaning of the poster with a partner. As much as possible, we tried to compose conversation dyads in such a way that all combinations of those who did and those who did not actually understand the message, as well as those who did and those who did not think they understood would be equally represented.
- *Phase 3*: Right after the conversations, we asked the conversation partners to individually fill in a second questionnaire almost identical to the phase 1 questionnaire.

### 4.2. Participants

A total of 200 young women aged between 13 and 17 years were randomly selected to take part in phase 1 of the study. All participants were second or third language English speakers, from schools with a student population of more than 500 students. From this group of 200 participants, 118 took part in phases 2 and 3 of the experiment (Table 1).

**Table 1.** Number of participants and dyads in the four schools.

	School				Total
	1	2	3	4	
Phase 1 participants	50	50	50	50	200
Phase 2 dyads	8	17	15	19	59
Phase 3 participants	16	34	30	38	118

We purposively only selected young women for this experiment, because we wanted to focus on one of the most vulnerable groups with regard to the theme of this study. Statistics indicate that in the teenage population in South Africa, the estimated HIV prevalence among them is eight times more than that of young men who are their counterparts. According to Shisana, Rehle, Simbayi, Zuma, Jooste, Zungu *et al*. (2014), an important explanation for this difference is that young women aged 15–19 years are more likely to have sex, not with their peers, but with older men. One-third (33.6%) of them reported having had a partner more than five years their senior, compared to only 4.1% of the young men (67–69). These ‘intergenerational relationships’ constitute one of the important factors contributing to HIV. In selecting women only, we also wanted to exclude mixed gender dyads in this first exploratory conversational study, because sexually related topics are still considered a taboo among most African cultures in discussions. This is especially true in those held across genders and different age groups (Baxen & Breidlid 2009; Bwanali 2008).

### 4.3. Materials

#### 4.3.1. Poster

**Fig. 1.**
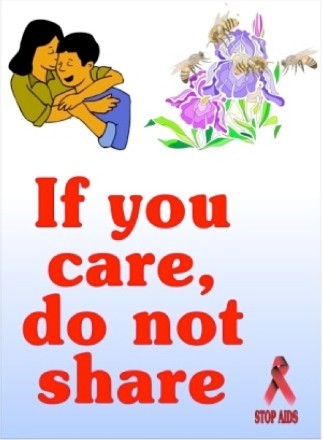
The poster used in this study.

The poster selected for this study is presented in Fig. 1. It was one of 16 posters used in a previous study into the effects of rhetorical figures in HIV and AIDS messages among young South Africans (Lubinga *et al*. 2014). In that study, we observed that there was a very low general level of AC of the messages. We decided to choose a poster that scored relatively high on AC (*M* = 2.36, SD = 1.09 on a scale of 1–4), and also relatively high on reported willingness to discuss it among peers (*M* = 3.37, SD = 1.00, on a scale of 1–4). The poster was in English because the results of a previous study conducted on the comprehension of puzzling messages in two African languages and English indicated that there were no differences in comprehension by participants on the basis of the message languages (Lubinga & Jansen 2011). The poster included the verbal message, ‘If you care, do not share’, plus a combination of pictures: a couple hugging, a single flower with several bees hovering over it, and a red ribbon underneath it with the text, ‘Stop AIDS’. The intended message was that having sexual relations with more than one partner at the same time could lead to getting HIV and AIDS. In an earlier study (Lubinga *et al*. 2010) comprehension of existing puzzling posters and radio messages was tested. Informal observations in that study learned that some of the participants were already familiar with these messages. In order to prevent that in the present study conversations would be affected by prior knowledge, it was decided not to use a puzzling message from an existing health campaign but to use a poster that was constructed such that it was as similar as possible to those that are used in campaigns.

#### 4.3.2. Questionnaires

We used two almost identical questionnaires in this experiment to measure AC, PC, and relevant beliefs, both before (phase 1) and after (phase 3) the conversation. The questionnaire that we presented in phase 1 was also used to select dyads for the discussions in phase 2. The questionnaire was piloted prior to the final data collection at a high school, different from the ones in the final study. During the pilot studies the researchers also tested the feasibility of the three-phased process among high school learners of the same age group as the participants in the final study. The questionnaires were in English. The decision to use questionnaires in English follows a previous study in which interviews in two mother-tongue languages and English were used to find out the comprehension of puzzling HIV/AIDS messages (Lubinga & Jansen 2011). The results of that study did not indicate better comprehension, nor preferences among a similar group of learners for asking the questions in their mother-tongues. Furthermore, the pilot studies of the present study ensured that the English questions were tailored to the understanding of the learners.

AC was measured by one open question: ‘Can you explain the most important message that this poster is trying to give to you?’ Participants were asked to write down their answer on the same page.

For own PC, we used one closed question: ‘How easy do you find it to understand this poster?’ followed by a 4-point scale (1 very difficult to understand, to 4 very easy to understand). We asked the participants to explain their choice in an open-ended question, which followed every closed one. The same question format was used to measure PC by friends and elder people, perceived willingness to discuss the poster with friends and elder people, and perceived personal relevance of the message.

We measured beliefs using seven statements, followed by 4-point scales (1 completely disagree to 4 completely agree) and an open-ended question asking the participants to explain their choice. The statements addressed HIV and AIDS-related themes. Two of the statements directly related to the message of the poster: ‘For a man, it is acceptable to have more than one girlfriend’ and ‘For a woman, it is acceptable to have more than one boyfriend'. The other five statements were as follows: ‘Abstinence is the best option in HIV prevention’; ‘For unmarried women, virginity is important’; ‘It is a good idea to combine medicine from the *sangomas* [witchdoctors] with medicine from the medical doctors’; ‘For unmarried men, virginity is important’; ‘AIDS can be cured by having sexual relations with a young child or a virgin.’

Personal questions were asked about age, gender, home language, other languages, profession of father and mother. The last question in phase 1 was a filler question: ‘What is the most interesting conversation you have had during the past few months?’ This question was included to keep participants in the classroom while the experimenter was assessing the AC so as to determine the dyads for phase 2.

#### 4.3.3. Procedure

The Provincial Department of Education and heads of the schools gave us permission to conduct the experiment, prior to the study. During the experiment, the participants were informed orally and in writing about the study and about their right to decline to participate or to discontinue their participation at any time. A small number of participants indeed decided to stop participating after phase 1 because they were uncomfortable or had other commitments.

We carried out two pilot studies in secondary schools different from those where the main experiment was carried out. The pilot studies aimed at finding the best composition for the conversations (dyadic or triadic), at deciding on the duration of the conversations, at testing the logistics of conducting this three-phased study on one day for each of the schools, and also at testing the questionnaire (see above). On the basis of the experiences thereof, we observed that many participants were not fluent English language speakers, and could not conduct conversations for a long period. We therefore decided to opt for dyadic 2-minute conversations in the main experiment. Such a short time frame does not seem unrealistic for conversations on a health topic. Boone and Lefkowitz (2007), for instance, found that during conversations of dyads of mothers and adolescents who were requested to discuss a health topic on ‘health’ and on ‘future’ for 7 minutes, the mean number of seconds that the mothers spent discussing on-topic material were quite low (range: 0.74–2.64 seconds; 1042).^1^

In the main experiment in each school, we scheduled the three phases on the same day as follows. Phase 1 took place in one room and took approximately one hour. The participants spent about 30–40 minutes filling in the questionnaire. The rest of the time was used to select the dyads. Phase 2 took place in parallel sessions with one of the three research assistants accompanying each dyad. The time needed for phase 2 depended on the number of dyads (differing per school) and took an hour or less, depending on the number of dyads per school. Phase 3 took place in one room again; it took the participants about 30 minutes to complete the questionnaire. We repeated the procedure below in each of the four schools.

In phase 1, we took 50 participants to a quiet classroom and asked all of them to sit on individual tables, all facing in the same direction. The researchers (the project leader/first author and three research assistants) introduced themselves to the participants and gave them oral and written instructions about the procedure of the experiment. For ease of identification, necessary for the subsequent phases, each participant was given a number tag, from 1 to 50, before filling in the questionnaire. Next, they each received the questionnaire on paper, stapled in such a way that the first two questions (about AC and own PC) could easily be separated from the rest. We requested them to raise their hand once they had completed these questions and that on completion of the entire questionnaire, each should wait quietly in their seat.

After the instructions, the participants started filling in the questionnaire. Once a participant raised her hand, a research assistant collected the answers to the first two questions and handed them to the project leader. She started registering the AC and PC and composing the dyads for phase 2 (see below) while the participants continued with the rest of the questionnaire. A research assistant took those who had been selected for phase 2 to the next venue.

In phase 2, we tried to compose a completely balanced set of dyads. However, this proved not to be possible. The final composition of dyads was as follows: 21 dyads with both identical AC statuses and identical PC statuses (both partners understood and thought they understood the message; both partners understood but thought they did not understand the message; both partners did not understand but thought they understood the message; both partners did not understand and thought they did not understand the message); 38 dyads with different AC and PC statuses (for instance, one partner understood and thought she understood the message whereas the other partner did not understand and thought she did not understand the message). Furthermore, the dyads were composed of partners who were from the same grade in each of the schools.

Once we had selected the dyads, we thanked the other participants for their participation, gave them a non-monetary incentive and requested them to leave, without communicating with their peers. We took the selected dyads (*n* = 59, see Table 1) one at a time to another venue to commence with the conversations. For the conversations, a room was equipped with a table and two chairs and a video camera installed so that the conversation could be recorded. The participants had to wait until the previous conversation was finished. At entering the room, we asked the pair to sit facing each other, sharing a table on which a copy of the poster was placed for each of them, in view of the video camera. We gave them the following instruction: ‘In front of you is the poster that you have just answered questions about. Please discuss the meaning of the poster message with your partner, in two minutes.’ We informed them too that the conversations were video and audio taped. After the instruction, we left the room and stood outside the door, within earshot but out of sight of the participants, to allow for a more natural flow of the conversation. We only intervened by entering the room and requesting the conversation partners to continue with their discussion if they had not talked to each other for about 30 seconds. We stopped the conversation and the recording after two minutes of conversation. A research assistant took the conversation partners to a venue at which phase 3 took place, in a joint session for all the participants.

In phase 3, after the conversations, we asked the participants to individually fill in a questionnaire at a new venue. We informed them that this questionnaire was almost identical to the one they filled in during phase 1. On completion, we gave them an incentive and requested them to go home.

## 5. Data analysis

### 5.1. Coding AC

We coded the AC of phase 1 participants (*n* = 200) provisionally during phase 1 of the experiment, just to enable the project leader to compose as many types of dyads as possible. After the experiment, two of the authors of this article scored the AC of the selected participants (*n* = 118) before and after the conversations as correct or incorrect. This resulted in a correctness score for one’s AC before the conversation (bAC: correct or incorrect) and after (aAC: correct or incorrect). We considered an interpretation as correct if it expressed the idea that sleeping with many partners might lead to HIV and AIDS (see the definition in the semantic analysis below). After a first coding round, we discussed scoring differences. We then decided to also include as correct other cases of ‘sharing’ in the context of HIV and AIDS, such as sharing needles, or sharing blood with another person. The third author coded all results with the same coding instructions as used by the first two authors. There were no differences between bAC coding from the first and the second author, and the third author. For AC after the conversations (aAC) scores, 10 (out of 118) cases of coding differed (Cohen’s kappa = .83). After discussion we solved all these cases and then used the results to define the final distribution of AC statuses of the dyads as used in the analysis.

### 5.2. Coding perceived own comprehension

The project leader also coded PC during phase 1, to compose dyads of various AC and PC statuses. For reasons of transparency and in view of the uneven distribution of the scores on the 4-point scales that were used, we recoded the scores of the selected participants into binary scores for PC before (bPC: low or high) and after the conversation (aPC: low or high). We then used these scores in the statistical analyses to define the final distribution of PC of the dyads.

### 5.3. Segmentation of conversations into information units

A research assistant transcribed verbatim the video recordings of all the conversations, then the project leader verified all the transcripts. Thereafter we divided all of them into information units (*n* = 1005, an average of 8.52 per participant). We also defined them using the following criteria on the level of conversation, content, syntax and prosody.

A unit is restricted to one conversational turn of one participant, but one conversational turn can consist of more than one unit; it is a meaningful utterance about one topic; it has the form of a full or contracted phrase, clause or sentence. Juxtaposed sentences are considered as different units. Subordinate clauses are only considered as separate units if there is a long pause or if there is a lack of topic coherence with the main clause.

Even with these criteria, not all units could be segmented; as the content of some units was unclear, conversational turns were incomplete or were just interactional in nature. As an example, Excerpt 1 offers the transcription of a conversation between two partners with no AC and low PC before the conversation. It shows a number of interactional (1, 16, 19, 22) and incomplete (17) units. The other, ‘meaningful’, units not only represent full sentences or clauses (2, 3, 7, 12, 20) but also contracted sentence parts that continue preceding turns (5, 8, 9) or sentences with incomplete starts of a new sentence attached (4, 13).

**Excerpt 1.** Part of a conversation between two participants who before the conversation and after their conversation neither actually understood the message (bAC incorrect) nor thought they understood the message (bPC low).

1	p1	thoma [‘start’ in Sepedi]
2	p2	Hm, uh … [Researcher interruption] I am writing about two people that have in relationship between the love
3	p1	Yeah, I think um these people have maybe HIV and AIDS
4	p1	and they share this carrier then I think maybe this, this um, may have a …
5	p2	AIDS [supplies]
6	p1	have AIDS then … [continues]
7	p2	so how can we eh, protect them?
8	p1	maybe condom, they use condom
9	p2	or abstain
10	p1	yeah, or prevent
11	p2	or using condom so umm
12	p2	two people are boy and girls so they are in relationship about love
13	p2	so um if I am not wrong eh, this girl is HIV and AIDS so eh, they have eh, the …
14	p1	her boyfriend [supplies, while pointing at the poster]
15	p2	her boyfriend,
16	p1	yeah
17	p2	so eh …
18	p1	they love him
19	p2	yes so …
20	p1	and they cry,
21	p2	because of this HIV and AIDS
22	p1	Yeah

### 5.4. Categorisation of information units

We were first of all interested in the number and distribution of units testifying a correct interpretation of the poster message. The first two authors independently defined the correct units and explored other categories of units. They finally defined four major classes of units (see Table 2).

**Table 2.** Occurrence of the four main classes of units.

Units (*n* = 1005)	%
Correct and on-topic (*n* = 155)	15.4
Incorrect, off-topic or vague units (all harmless) (*n* = 697)	69.4
Incorrect and dangerous (*n* = 3)	0.3
Other (*n* = 150)	14.9

#### 5.4.1. Correct, on-topic units

Correct, on-topic units expressed the intended message: sharing sexual partners will lead to getting HIV and AIDS. The basic idea could be expressed more or less literally, for example, ‘I understand that if I have a partner … shouldn’t share him with anyone’, ‘And to stop sharing a boyfriend’, ‘When you are dating do not share’, ‘You do not go around and spread this disease to them.’ The idea could have been said in a more abstract fashion, for example, ‘Be faithful’, ‘I think the message is saying, be faithful to your partners, do not cheat’, ‘You must love one person.’ We also included a handful of units in which the crucial negative valence of ‘sharing’ was expressed, but not in relation to multiple partners, for example, ‘It means that you do not have to share things that can make you have an HIV and AIDS’, ‘Do not share needles with somebody.’

#### 5.4.2. Incorrect, off-topic or vague units (all harmless)

The range in the category of incorrect, off-topic or vague units (all harmless) was broad and entailed four different subclasses, each including clear as well as borderline cases.

*Incorrect, descriptive units* expressed what was visible in the poster, without expressing the intended message, for example, ‘Ok, so I see a man and a woman hugging, hugging someone here, so I don’t know’, ‘Ok when I see the message it says if you care do not share and it has the sign of stop AIDS.’ Many of these units included a repetition of (part of) the words on the poster, and the participants played around with these words. Sometimes participants shared this word play in subsequent units (/) in different turns (e.g. p1 ‘If you care do not care’/‘I mean if you care, do not share’/p2 ‘No, you cannot share because …’/p1 ‘yeah, but it says, If you care do not share’/p2 ‘I can care for you but don’t share with you’ …).

*Incorrect units showing the wrong interpretation of ‘sharing’* expressed the idea of sharing with a positive valence, neglecting the intended message, for example, ‘The message says you can share something like your secrets with your friend’, ‘You have to share with a friend’, ‘Hmm and we can also share food with her’, ‘Share it means that it is talking about love.’

In *off-topic units* participants raised many different AIDS- and HIV-related topics, for instance, disclosure of HIV status, pregnancy, abstinence, taking HIV medication, using condoms, or caring for those who are HIV positive, for example, ‘I think we should take care of others who have HIV/AIDS’, ‘Like, do not sleep with a boy without using a condom’, ‘Yes all the times we must know our status.’

*Vague units* were vague statements or combinations thereof, for example, ‘Ha, if you do not share, hape [again, in Sepedi] if you don’t share uh, uh, uh the couple might up, getting up failing or getting ill’, ‘And what we should do is that, we should make the right decision’, ‘And the way I understand this is that ummm, it helps many people to understand that there are so many things happening in life.’

#### 5.4.3. Incorrect and dangerous units

In view of the potential danger of wrong interpretations, we specifically looked for statements that would reveal a dangerous misinterpretation of the intended message, for example, ‘If you share one man, you might not get HIV because he might not have it’, and ‘HIV is not come from sleeping with someone.’

#### 5.4.4. Other units

Two types of units were found without any topical content:

*Interactional units* only related to the interaction between partners, such as invitations to speak, short utterances expressing agreement, or questions meant to solicit agreement or interpretation, for example, ‘Do you agree with me?’ or ‘What do you think?’, ‘Yeah’, ‘Hmm’.

*Incomplete units* were terminated because the partner took over with a new turn, for example, ‘I see this picture is, this picture is eeh …’

In developing the categories, the first two authors coded the units in different rounds and discussed the differences between them. Some subclasses of incorrect units remained difficult to distinguish, for example, the difference between off-topic and vague. As this difference was not crucial for our purposes, we took off-topic and vague units together in one class. Using the four categories in Table 2, the third author recoded about 30% of the units (*n* = 365). The percentage of units agreed on was high: 96.2 (level of agreement: Cohen’s kappa .92). Given this level of agreement, we decided to use the original coding by the first two authors for further analysis.

## 6. Results

### 6.1. Effects of conversations on AC

In order for us to determine how the learner's AC of the message after the conversation (aAC) was related to both the learner's own AC before the conversation (bAC) and the partner's AC before the conversation (pbAC), we performed two crosstab analyses.

The first crosstab analysis was carried out to assess how the correctness of the AC of the learners after the conversations (aAC) was related to their own AC before the conversations (bAC). Table 3 shows that the number of participants with a correct interpretation after the conversations happened to be the same as the number of participants having a correct interpretation before the conversations (*n* = 34). However, 30 changes in one’s AC status took place, and AC status after the conversation proved to be significantly affected by AC status before the conversation (chi square (df = 1) (Yates Continuity Correction) = 15.26; *p* < .001). The discussions were not really beneficial for this group of learners: 44.1% (15 out of 34) of the participants with a correct interpretation before the conversation ended up with an incorrect interpretation after, whereas only 17.9% (15 out of 84) of the participants changed the other way around: from an incorrect interpretation before to a correct interpretation after the conversation.

**Table 3.** Learners’ AC status after the conversations in relation with AC status before the conversations.

	aAC correct	aAC incorrect	Total
bAC correct	19	15	34
bAC incorrect	15	69	84
Total	34	84	118

The second crosstab analysis assessed how the AC of the learners after the conversations (aAC) was related to the AC of their conversation partner before the conversations (pbAC). As Table 4 shows, one’s AC status after the conversation was significantly affected by the AC status of one’s conversation partner’s (chi square (df = 1) (Yates Continuity Correction) = 4.46; *p* = .03). Having a conversation partner with a correct interpretation of the message proved to be beneficial. Of the participants with a partner having a correct interpretation (*n* = 34), 44.1% (*n* = 15) interpreted the message correctly after the conversation, whereas only 22.6% (*n* = 19) of the 84 participants with a partner having an incorrect interpretation interpreted the message correctly after the conversation.

**Table 4.** Learners’ AC status after the conversations in relation with partners’ AC status before the conversations.

	aAC correct	aAC incorrect	Total
pbAC correct	15	19	34
pbAC incorrect	19	65	84
Total	34	84	118

We carried out separate analyses for the participants with a correct or an incorrect AC before the conversation to determine to what extent the learners’ AC status before the conversations and their conversation partners’ AC status before the conversations interacted in influencing the AC status of the learners after the conversations (see Tables 5 and 6).

**Table 5.** For learners with correct bAC (*n* = 34): AC status after the conversations in relation to partners’ AC status before the conversations.

	aAC correct	aAC incorrect	Total
pbAC correct	5	1	6
pbAC incorrect	14	14	28
Total	19	15	34

**Table 6.** For learners with incorrect bAC (*n* = 84): AC status after the conversations in relation to partners’ AC status before the conversations.

	aAC correct	aAC incorrect	Total
pbAC correct	10	18	28
pbAC incorrect	5	51	56
Total	15	69	84

As Table 5 shows, for participants who interpreted the message correctly before the conversation (*n* = 34), AC status after the conversation did not prove to be significantly affected by the conversation partner’s AC status before the conversation (Fisher’s exact test: Phi = −.16; *p* = .20). As Table 6 shows, however, for participants who interpreted the message incorrectly before the conversation (*n* = 84), AC status after the conversation was significantly affected by the conversation partner’s AC status before the conversation (chi square (df = 1; Yates Continuity Correction) = 7.40; *p* < .01): 35.7% (*n* = 10). Of the 28 participants with a partner having a correct interpretation 35.7% (*n* = 10) interpreted the message correctly after the conversation, whereas only 8.9% (*n* = 5) of the 56 participants with a partner who interpreted the message incorrectly before the conversation interpreted the message correctly after the conversation.

Finally, it was assessed if the *difference* between one’s own AC before the conversation and after the conversation might be affected by one’s partner’s AC before the conversation. As Table 7 shows, the partner’s AC before the conversation had a positive effect on the development of one’s own AC (chi square (df = 2) = 14.37; *p* = .001).

**Table 7.** Difference between learners’ AC status after and before the conversations in relation with their partners’ AC status before the conversations.

	Positive	None	Negative	Total
pbAC high	10	23	1	34
pbAC low	5	65	14	84
Total	15	88	15	118

The results presented in Tables 3–7 can be summarised as follows:

- The comprehension of poster messages does not naturally benefit from conversations about their meaning. On the contrary, it is more probable that a correct interpretation before the conversation turns into an incorrect interpretation after the conversation than the other way around (Table 3).
- Having a conversation partner with a correct message interpretation helps. It is more probable for a learner with a well-informed conversation partner to end up with a correct interpretation of the message, than for a learner with a conversation partner who has an incorrect interpretation (Table 4). This effect was specifically found for learners with an incorrect interpretation before the conversation (Tables 5 and 6). Furthermore, the difference in AC after and before the conversation was found to be positively related with one’s partner’s AC before the conversation (Table 7)

### 6.2. Effects of conversations on PC

Two crosstab analyses were performed to determine how the learner's PC of the message (high or low) after the conversation (aPC) was related to the learner's own PC before the conversation (bPC) and to the partner's PC before the conversation (bPC).

The first crosstab analysis was carried out to assess how the learners’ perceived correctness of their interpretation after the conversations (aPC) was related to their perceived correctness before the conversations (bPC). As Table 8 shows, the conversations had a positive effect on PC: the number of participants with a high PC is higher after the conversations than before (*n* = 81 vs. *n* = 67). One's PC status after the conversation proved to be significantly affected by one's PC status before the conversation (chi square (df = 1) (Yates Continuity Correction) = 14.51; *p* < .001): 49% (25 out of 51) of the participants with a low PC before, changed into high PC, whereas 16.4% (11 out of 67) of the learners with a high PC before changed into low PC.

**Table 8.** Learners’ PC status after the conversations in relation with their PC status before the conversations.

	aPC high	aPC low	Total
bPC high	56	11	67
bPC low	25	26	51
Total	81	37	118

The second crosstab analysis was carried out to assess how learners’ perceived correctness of their interpretation after the conversations was related to their partners’ perceived correctness of their interpretation before the conversations. Table 9 shows that PC status after the conversation was not significantly affected by the conversation partner’s PC status before the conversation (chi square (df = 1) (Yates Continuity Correction) = 1.01; *p* = .31): 73.1% (49 out of 67) of the learners with a high PC partner had a low PC after the conversations, whereas 62.7% (32 out of 51) of the learners with a low PC partner had a high PC after the conversation.

**Table 9.** Learners’ PC status after the conversations in relation with their partners’ PC status before the conversations.

	aPC high	aPC low	Total
pbPC high	49	18	67
pbPC low	32	19	51
Total	81	37	118

Separate analyses were carried out for the participants with high or low PC before the conversation to determine to what extent the learners’ PC status before the conversations and their conversation partners’ PC status before the conversations interacted in influencing the PC status of the learners after the conversations (see Tables 10 and 11). In none of these groups, was PC status after the conversation significantly affected by the conversation partner’s PC status before the conversation (for the bPC high group: Fisher’s exact test: Phi = −.11; *p* = .45; for the bPC low group: chi square (df = 1) (Yates Continuity Correction) = 1.19; *p* = .27).

**Table 10.** For learners with a high PC before the conversations (*n* = 67): PC status after the conversations in relation to their partners’ PC status before the conversations.

	aPC high	aPC low	Total
pbPC high	43	7	50
pbPC low	13	4	17
Total	56	11	67

**Table 11.** For learners with a low PC before the conversations (*n* = 51): PC status after the conversations in relation to their partners’ PC status before the conversations.

	aPC high	aPC low	Total
pbPC high	6	11	17
pbPC low	19	15	34
Total	25	26	51

The results presented in Tables 8–11 can be summarised as follows.

- It is more probable that a conversation leads to a change from low to high PC than to a change from high to low PC (Table 8).
- Having had a conversation partner with a high PC was not found to affect the learner's own PC after the conversation (Table 9), neither in learners who before the conversation thought they understood the message, nor in learners who before he conversation thought they did not understand the message (Tables 10 and 11).

### 6.3. Effects of conversations on the relationship between AC and PC

Two crosstab analyses were performed to determine how the conversations affected the relationship between participants’ AC and their PC (see Tables 12 and 13).

**Table 12.** Learners’ PC status before the conversations in relation to their AC status before the conversations.

	bPC high	bPC low	Total
bAC correct	23	11	34
bAC incorrect	44	40	84
Total	67	51	118

**Table 13.** Learners’ PC status after the conversations in relation to their AC status after the conversations.

	aPC high	aPC low	Total
aAC correct	25	9	34
aAC incorrect	56	28	84
Total	81	37	118

As Table 12 shows, the number of participants whose interpretation of the message before the conversation was correct (*n* = 34) is lower than the number of participants who thought their interpretation before the conversation was correct (*n* = 67). No significant relation was found between the perceived and the actual correctness of the interpretation (chi square (df = 1) (Yates Continuity Correction) = 1.72; *p* = .19).

As Table 13 shows, the number of participants whose interpretation of the message after the conversation was correct (*n* = 34) is lower than the number of participants who thought their interpretation after the conversation was correct (*n* = 81). Again, no significant relation was found between the perceived and the actual correctness of the interpretation (chi square (df = 1) (Yates Continuity Correction) = 0.26; *p* = .61).

Comparing the results in Tables 12 and 13 reveals no clear effect of the conversations on the relationship between participants’ AC and their PC: neither before the conversations nor after the conversations there was a statistically significant relation between a learner's perception that her interpretation was correct and the actual correctness of her interpretation. However, whereas the number of learners with a correct interpretation did not change as a result from the conversations (bAC correct = 34 and aAC correct = 34), the number of learners who thought their interpretation was correct grew with 21% (bPC high = 67; aPC high = 81).

### 6.4. Effects of conversations on beliefs

The beliefs with respect to the topic of the poster message (i.e. multiple relationships) before and after the conversation were compared in two paired samples *T*-tests (*n* = 117; one missing value). For the belief that ‘For a man, it is acceptable to have more than one girlfriend’, the conversations turned out to have a negative effect. On average, this unwanted belief was stronger after the conversations than before: *M*_after_ = 2.10, SD = 1.35; *M*_before_ = 1.80, SD = 1.18; *t*(116) = 2.07; *p* = .04 (two-sided). No significant effect of conversations was found for the belief that ‘For a woman, it is acceptable to have more than one boyfriend’: *M*_after_ = 1.76, SD = 1.26; *M*_before_ = 1.88, SD = 1.26; *t*(116) = −0.92; *p* = .36 (two-sided).

One significant effect of the conversations was found for one of the other beliefs that were measured. The mean score for the belief about combining medicines (‘It is a good idea to combine medicine from the *sangomas* with medicine from the medical doctors’) was higher after the conversations than before: *M*_after_ = 2.09, SD = 1.35; *M*_before_ = 1.86, SD = 1.26; *t*(116) = 2.04; *p* = .04 (two-sided).

### 6.5. Effects of AC and PC on the quality and quantity of the contributions to the conversations

#### 6.5.1. AC and the quality of contributions

To establish the quality of contributions to the conversations from learners who understood the message before the conversations compared to learners who did not, a multivariate analysis of variance (MANOVA) was conducted assessing the effects of the independent variable 'correctness of AC of the message before the conversation' (bAC: correct vs. incorrect) on four dependent variables: the participants’ numbers of contributions that were considered as (a) 'correct and on-topic', (b) ‘incorrect, off-topic or vague (all harmless)’, (c) ‘incorrect and dangerous’, or (d) 'other'. The MANOVA revealed a significant multivariate effect of bAC: Wilk’s Lambda = .80; *F*(4, 113) = 7.22, *p* < .001; *η*^2^ = .20. Follow-up univariate analyses showed a significant effect of bAC on (a) correct and on-topic (*F*(1, 116) = 25.04, *p* < .001; *η*^2^ = .18). On average, the participants who showed the correct interpretation of the message before the conversation contributed more correct and relevant units (*M* = 2.47, SD = 2.31) than the participants who showed an incorrect interpretation of the message before the conversation (*M* = 0.81, SD = 1.27). We found no significant effects on any of the other dependent variables.

#### 6.5.2. PC and the quality of contributions

We also conducted a MANOVA to find possible effects of PC before the conversation on the quality of the contributions from the participants, with ‘PC of the message before the conversation’ (bPC: high vs. low) as independent variable, and the participants’ numbers of contributions that were considered as (a) 'correct and on-topic', (b) ‘incorrect, off-topic or vague (all harmless)’, (c) ‘incorrect and dangerous’, or (d) 'other' as dependent variables. No significant multivariate effect of bPC was found: *F*(4, 113) = 1.78, *p* = .14.

#### 6.5.3. PC and the quantity of contributions

It was expected that learners with high PC would be more confident and thus productive in the conversations, we compared the average number of words and units produced by learners with a high and low PC before the conversation. Two one-way between subjects ANOVA's with 'PC before the conversation' (bPC: high vs. low) as independent variable and 'number of units uttered by participant' and 'number of words uttered by participant' as dependent variables. No significant effects were found.

On average, the participants who had a correct bPC, contributed more correct, on-topic contributions to the conversations than those who did not. It should be noted however, that the overall informational quality of the conversations was low. The total percentage of correct, on-topic units was only 15.4% (see Table 2). PC did not affect the quality or quantity of contributions to the conversations.

## 7. Discussion

In this article, we studied conversations between young women in South Africa about a puzzling HIV and AIDS related campaign message. We investigated the effect of their AC and PC, the relationship between these two, their beliefs about the message topic and the extent to which bAC and bPC affected the quality and the quantity of the participants’ contributions to the conversations.

### 7.1. Conversations and message comprehension

The results show that conversations about the meaning of a puzzling message do not spontaneously improve participants’ AC. On the contrary, it was even more probable that a correct interpretation of a puzzling message before the conversation would turn into an incorrect interpretation after the conversation than the other way round. Our study also reveals the importance of having a knowledgeable partner. Participants with such a partner had a higher chance of acquiring a correct message interpretation during conversation than participants with a conversation partner who lacked an adequate interpretation of the poster message.

With regard to the role of PC, we know from previous studies that when individuals think that they understand the meaning of the message, they are more willing to talk about it (Hoeken *et al*. 2009; Jansen & Janssen 2010; Lubinga & Jansen 2011; Lubinga *et al*. 2010, 2014). The current study contributes to this line of work in that it shows that conversations strengthen the confidence of the participants that they understood the message. More participants with a low PC before the conversation had a high PC after the conversation than the other way around. Changes in one’s PC were not found to be affected by the prior PC status of the conversation partner, neither for the participants who thought that they had understood the message before the conversation nor for those who thought that they had not understood it.

We also wanted to find out whether the relationship between PC and AC would be different before and after the conversation. On the whole, the results both before and after the conversations support the conclusions of Jansen and Janssen (2010) and Lubinga *et al*. (2010). There were more participants who thought they understood the message than participants who actually did. The number of participants who perceived their comprehension as correct even increased after the conversation, whereas the number of those with a correct AC remained constant. This suggests that conversations might strengthen the illusion in receivers of a puzzling message that they understand the meaning, when the reality is that they do not. The unintended result of conversations could then be that the recipients mistakenly gain confidence in their incorrect interpretations. This finding is important for media campaign designers who construct complex messages with the intention of triggering conversations about the topics addressed in the messages. Such messages can lead to dangerous confusion and misunderstanding of health risks and risk prevention (see also Cho & Salmon 2007; Fishbein & Yzer 2003; Hoeken *et al*. 2009). Fortunately, in our study we hardly found any conversation units that indicated dangerous misinterpretations of the intended message.

### 7.2. Conversations and beliefs

We asked the participants to rate seven beliefs before and after the discussions, five of which represented undesirable behaviour. Two of these undesirable beliefs related directly to the poster message. For one of the message-related ones, as well as for one of the five non-message related ones, the scores after the conversation were significantly higher than before. This increase in scores for undesirable beliefs could have been caused by no more than a mere recognition effect due to re-reading and recognising the same belief statements in both the first and second questionnaire. However, another, more disturbing explanation could be that conversations, especially among partners who are both not very knowledgeable might indeed result in strengthening undesirable beliefs. In any case, our results pertaining to comprehension and beliefs should make one cautious about positive results of conversations found in earlier studies.

For example, our belief results clearly contrast with the findings in Hwang (2012:14). In that study, Hwang found that higher levels of campaign conversation led to stronger antismoking beliefs. It should be noted that the conversations in Hwang (2012) were combined with other campaign interventions that could have also influenced the beliefs that were measured. Others like Chandler, Canty-Mitchell, Kip, Daley, Morrison-Beedy, Antsey *et al*. (2013) or Lefkowitz *et al*. (2004) also suggest that conversations result into more positive intentions and beliefs. Our results show otherwise. Perhaps this difference may partly be explained by the overall low level of comprehension of the puzzling message that we used in our study. New studies could lead to more clarity about this.

### 7.3. Quality and quantity of contributions to the conversations

In general, we found that participants, who had a correct understanding of the message prior to the conversation, had better quality contributions to the discussions in terms of the relevance to the topic of the message, than those who did not. There was no effect of PC on the quality or the quantity of contributions made to the conversations. The overall quality of the conversations was low. Only about 15% of the contributions expressed the intended message in the poster. A low individual knowledge status seems to lead to a low quality of conversations, which in turn negatively affects the final interpretation of the message, thereby suggesting a bi-directional relationship between the knowledge status and the conversation quality.

## 8. Limitations of this study

This study is not without its limitations. First, it is difficult to generalise the findings to real world, natural conversations. We explicitly instructed participants to talk about the message in order to create a controlled environment in which it could be ensured that the participants would start a conversation about the message presented to them, and would stay on-topic for two minutes.

Second, all our participants were young women from a rural part of one province in South Africa, their proficiency in English was limited, and they generally had a low level of comprehension of the message presented to them. Lubinga and Jansen (2011), though, found that the use of English or an African language in puzzling health messages or in interviews about these messages did not affect comprehension scores from mother tongue speakers of the African language involved. However, in contrast to the task for the participants in Lubinga and Jansen (2011), who were interviewed by a researcher, participants in the present study were requested to conduct conversations among each other in English. If they would have been asked to conduct their conversations in their first language, results could have been different. Further studies could explore the effects of conversations conducted in the participants’ mother tongue.

Third, there was only one health-related topic that we asked the participants to discuss. Replication studies are needed to test if the same type of outcomes would also be found in different research populations composed of, for instance, mens’ or mixed gender dyads, adults, living in urban areas and/or in another country, fluent in English, and/or with a better understanding of similar or other messages than used in our study.

Finally, one might argue that the duration of the conversations in our study is low: 2 minutes, and that no more than 15% of the contributions were topic relevant. However, compared to Boone and Lefkowitz’s (2007) study, where one of the partners never spent more than 0.3% of the 7 minutes of conversation time on health topics, in our study the proportion of relevant contributions should be considered as relatively high.

## 9. Conclusion

To our knowledge, this study is the first to explore in detail the content of conversations about a mass media health message, in this case a message that was not easy to understand for many receivers in the target group. We found some provocative results. The conversations led to a lower rather than a higher understanding of the message, to a greater rather than a smaller discrepancy between what the receivers thought they understood and what they really understood, and to an increase rather than a decrease of undesirable beliefs about the topic of the message. Although conversations among peers might be valuable in health campaigns, our study shows that intended positive effects do not automatically follow.

Our findings have implications for health campaign designers who advocate for the use of puzzling messages to provoke conversations with the intention of positively influencing (determinants of) health behaviour. Exposure to a puzzling health message does not automatically lead to conversations among targeted audience members that result in improved comprehension and beliefs. More research is needed in this field to help designers of mass media health campaigns to create messages that their audience finds interesting enough to discuss, but which do not at the same time lead to possibly dangerous misunderstanding and undesirable beliefs.

## **Note**

1. In personal communication held in October 2014, Dr Boone confirmed that the number of seconds (not minutes) that mothers spent discussing some of the health topics in their study, was indeed very low.

## References

[CIT0001] Arroyo A. & Harwood J. (2012). Exploring the Causes and Consequences of Engaging in Fat Talk. Journal of Applied Communication Research, 40(2), 167–187. doi: 10.1080/00909882.2012.654500

[CIT0002] Baelden D., Audenhove L. V. & Vergnani T. (2012). Using New Technologies for Stimulating Interpersonal Communication on HIV and AIDS. Telematics and Informatics, 29(2), 166–176. doi: 10.1016/j.tele.2011.05.002

[CIT0003] Baxen J. & Breidlid A. (Eds.). (2009). HIV and AIDS in Sub-Saharan Africa. Understanding the Implications of Culture and Context, Cape Town, UCT Press.

[CIT0005] Boone T. L. & Lefkowitz E. S. (2007). Mother-Adolescent Health Communication: Are All Conversations Created Equally? Journal of Youth and Adolescence, 36(8), 1038–1047. doi: 10.1007/s10964-006-9138-2

[CIT0006] Busse P., Fishbein M., Bleakley A. & Hennessy M. (2010). The Role of Communication with Friends in Sexual Initiation. Communication Research, 37(2), 239–255. doi: 10.1177/0093650209356393 20613973 PMC2897170

[CIT0007] Bwanali A. K. (2008). Language and HIV and AIDS. Openspace, 2(3), 64–70.

[CIT0008] Chandler R., Canty-Mitchell J., Kip K. E., Daley E. M., Morrison-Beedy D., Antsey E., et al. (2013). College Women’s Preferred HIV Prevention Message Mediums: Mass Media Versus Interpersonal Relationships. Journal of the Association of Nurses in AIDS Care, 24(6), 491–502. doi: 10.1016/j.jana.2012.09.001 23465402

[CIT0009] Chatterjee J. S., Bhanot A., Frank L. B., Murphy S. T. & Power G. (2009). The Importance of Interpersonal Discussion and Self-Efficacy in Knowledge, Attitude and Practice Models. International Journal of Communication, 3(2), 607–634.

[CIT0010] Cho H. & Salmon C. T. (2007). Unintended Effects of Health Communication Campaigns. Journal of Communication, 57(2), 293–317. doi: 10.1111/j.1460-2466.2007.00344.x

[CIT0011] Dillorio C., Kelley M. & Hockenberry-Eaton M. (1999). Communication about Sexual Issues: Mothers, Fathers, and Friends. Journal of Adolescent Health, 24(3), 181–189. doi: 10.1016/S1054-139X(98)00115-3 10195801

[CIT0012] Dunlop S. M. (2011). Talking ‘Truth’: Predictors and Consequences of Conversations about a Youth Antismoking Campaign for Smokers and Non-smokers. Journal of Health Communication, 16(7), 708–725. doi: 10.1080/10810730.2011.552000 21476165

[CIT0013] Dunlop S. M., Cotter T. & Perez D. (2014). When Your Smoking Is Not Just about You: Antismoking Advertising, Interpersonal Pressure and Quitting Outcomes. Journal of Health Communication, 19(1), 41–56. doi: 10.1080/10810730.2013.798375 23967804

[CIT0014] Dunlop S. M., Kashima Y. & Wakefield M. (2010). Predictors and Consequences of Conversations about Health Promoting Media Messages. Communication Monographs, 77(4), 518–539. doi: 10.1080/03637751.2010.502537

[CIT0015] Dunlop S. M., Wakefield M. & Kashima Y. (2008). The Contribution of Antismoking Advertising to Quitting: Intra- and Interpersonal Processes. Journal of Health Communication, 13(3), 250–266. doi: 10.1080/10810730801985301 18569357

[CIT0016] Fishbein M. & Yzer M. C. (2003). Using Theory to Design Effective Health Behavior Interventions. Communication Theory, 13(2), 164–183. doi: 10.1111/j.1468-2885.2003.tb00287.x

[CIT0017] Frank L. B., Chatterjee J. S., Chaudhuri S. T., Lapsansky C., Bhanot A. & Murphy S. T. (2012). Conversation and Compliance: Role of Interpersonal Discussion and Social Norms in Public Communication Campaigns. Journal of Health Communication, 17(9), 1050–1067. doi: 10.1080/10810730.2012.665426 22808934

[CIT0018] Frank L. B., Murphy S. T., Chatterjee J. S., Moran M. B. & Baezconde-Garbanati L. (2015). Telling Stories, Saving Lives: Creating Narrative Health Messages. Health Communication, 30(2), 154–163. doi: 10.1080/10410236.2014.974126 25470440 PMC5608451

[CIT0019] Geary C. W., Burke H. M., Castelnau L., Neupane S., Sall Y. B., Wong E., et al. (2007). MTV’s ‘Staying Alive’ Global Campaign Promoted Interpersonal Communication About HIV and Positive Beliefs About HIV Prevention. AIDS Education and Prevention, 19(1), 51–67. doi: 10.1521/aeap.2007.19.1.51 17411389

[CIT0020] Hafstad A. & Aaro L. E. (2009). Activating Interpersonal Influence Through Provocative Appeals: Evaluation of a Mass Media-Based Anti-smoking Campaign Targeting Adolescents. Health Communication, 9(3), 253–272. doi: 10.1207/s15327027hc0903_4

[CIT0021] Helme D. W., Noar S. M., Allard S., Zimmerman R. S., Palmgreen P. & McClanahan K. J. (2011). In-depth Investigation of Interpersonal Discussions in Response to a Safer Sex Mass Media Campaign. Health Communication, 26(4), 366–378. doi: 10.1080/10410236.2010.551582 21409674 PMC4529750

[CIT0022] Hendriks H., Van den Putte B. & De Bruijn G. (2014a). Changing the Conversation: The Influence of Emotions of Conversation Valence and Alcohol Consumption. Prevention Science, 15(5), 684–693. doi: 10.1007/s11121-013-0418-2 23812888

[CIT0023] Hendriks H., Van den Putte B., De Bruijn G. & De Vreese C. H. (2014b). Predicting Health: The Interplay Between Interpersonal Communication and Health Campaigns. Journal of Health Communication: International Perspectives, 19(5), 625–636. doi: 10.1080/10810730.2013.837552 24446759

[CIT0024] Hoeken H., Swanepoel P., Saal E. & Jansen C. (2009). Using Message Form to Stimulate Conversations: The Case of Tropes. Communication Theory, 19(1), 49–65. doi: 10.1111/j.1468-2885.2008.01332.x

[CIT0025] Hollemans E. (2005, January 18). loveLife Gets Attitude. Mail & Guardian. http://www.mg.co.za/article/2005-01-18-lovelife-gets-attitude (Accessed 1 May 2015).

[CIT0026] Hornik R. C. & Yanovitsky I. (2003). Using Theory to Design Evaluations of Communication Campaigns: The Case of the National Youth Anti-Drug Media Campaign. Communication Theory, 13(2), 204–224. doi: 10.1111/j.1468-2885.2003.tb00289.x 25525317 PMC4267481

[CIT0027] Hwang Y. (2012). Social Diffusion of Campaign Effects: Campaign-Generated Interpersonal Communication as A Mediator of Anti-toBACco Campaign Effects. Communication Research, 39(1), 120–141. doi: 10.1177/0093650210389029

[CIT0028] Jansen C. & Janssen I. (2010). Talk about It: The Effects of Cryptic HIV/AIDS Billboards. Communicatio, 36(1), 130–141. doi: 10.1080/02500160903525072

[CIT0029] Kam J. A., Potocki B. & Hecht M. L. (2012). Encouraging Mexican Heritage Youth to Intervene When Friends Drink: The Role of Targeted Parent-Child Communication Against Alcohol. Communication Research, 20(10), 1–21.

[CIT0030] Katz E. & Lazarsfeld P. (1955). Personal Influence, New York, Free Press.

[CIT0031] Khalil G. E. & Rintamaki L. S. (2014). A Televised Entertainment-Education Drama to Promote Positive Discussion about Organ Donation. Health Education Research. doi:10.1093/her/cyt106. PMC4481582 24399264

[CIT0032] Lefkowitz E. S., Boone T. L. & Shearer C. L. (2004). Communication with Best Friends about Sex-Related Topics During Emerging Adulthood. Journal of Youth and Adolescence, 33(4), 339–351. doi: 10.1023/B:JOYO.0000032642.27242.c1

[CIT0033] Lubinga E. & Jansen C. (2011). ‘No ‘Til We Know’ Fela ba A Tseba naa? The Influence of the Language of Communication on the Reception of HIV/AIDS Messages among Young South Africans. Communicatio, 37(3), 466–481. doi: 10.1080/02500167.2011.589394

[CIT0034] Lubinga E., Jansen C. & Maes A. (2014). ‘If You Care, Do Not Share’. Exploring the Effects of Using Rhetorical Figures to Stimulate Young South Africans to Discuss HIV and AIDS Messages. Communicatio, 40(1), 49–68. doi: 10.1080/02500167.2014.868365

[CIT0035] Lubinga E., Schulze M., Jansen C. & Maes A. (2010). HIV/AIDS Messages as A Spur for Conversation among Young South Africans? African Journal of AIDS Research, 9(2), 175–185. doi: 10.2989/16085906.2010.517487 25860526

[CIT0036] Nabi R. L. (2015). Emotional Flow in Persuasive Health Messages. Health Communication, 30(2), 114–124. doi: 10.1080/10410236.2014.974129 25470436

[CIT0037] Reimuller A., Hussong A. & Ennett S. T. (2011). The Influence of Alcohol-Specific Communication on Adolescent Alcohol Use and Alcohol Related Consequences. Prevention Science, 12(4), 389–400. doi: 10.1007/s11121-011-0227-4 21667141 PMC3816119

[CIT0038] Ritson M. & Elliot R. (1999). The Social Uses of Advertising: An Ethnographic Study of Adolescent Advertising Audiences. Journal of Consumer Research, 26(3), 260–277. doi: 10.1086/209562

[CIT0039] Robbins D. (2010). Beyond the Billboards. The LoveLife Story, Johannesburg, Porcupine Press.

[CIT0040] Rogers E. (1995). Diffusion of Innovations (4th ed.), New York, Free Press.

[CIT0041] Shisana O., Rehle T., Simbayi L. C., Zuma K., Jooste S., Zungu N., et al. (2014). South African National HIV Prevalence, Incidence and Behavior Survey, 2012, Cape Town, HSRC Press. 10.2989/16085906.2016.1153491 27002359

[CIT0042] Singer R. (2005, August 24). Is loveLife Making Them Love Life? Mail & Guardian. http://mg.co.za/article/2005-08-24-is-lovelife-making-them-love-life (Accessed 1 May 2015).

[CIT0043] Southwell B. G. (2005). Between Messages and People: A Multi-level Model of Memory for Television Content. Communication Research, 32(1), 112–140. doi: 10.1177/0093650204271401

[CIT0044] Southwell B. G. & Yzer M. C. (2009). When (and Why) Interpersonal Talk Matters for Campaigns. Communication Theory, 19(1), 1–8. doi: 10.1111/j.1468-2885.2008.01329.x

[CIT0045] Van den Putte B., Yzer M., Southwell B. G., De Bruijn G. & Willemsen M. C. (2011). Interpersonal Communication as an Indirect Pathway for the Effect of Antismoking Media Content on Smoking Cessation. Journal of Health Communication, 16(6), 470–485. doi: 10.1080/10810730.2010.546487 21337250

